# Consequences of academic disappointment inventory: confirmatory factor analysis, reliability and convergent validity

**DOI:** 10.1186/s40359-025-02610-6

**Published:** 2025-04-24

**Authors:** Ayşen Senem Çopur, Gyöngyi Kökönyei

**Affiliations:** 1 Doctoral School of Psychology, ELTE Eötvös Loránd University, Izabella Street 46, Budapest, 1064 Hungary; 2 Institute of Psychology, ELTE Eötvös Loránd University, Izabella Street 46, Budapest, 1064 Hungary; 3 NAP3.0-SE Neuropsychopharmacology Research Group, Hungarian Brain Research Program, Semmelweis University, Budapest, Hungary; 4 Department of Pharmacodynamics, Pharmaceutical Sciences, Semmelweis University, Budapest, Hungary

**Keywords:** Academic emotions, Academic disappointment, Student engagement, Emotions, Scale validation, Educational psychology

## Abstract

**Background and aims:**

The research literature on academic disappointment and its relationship to student engagement is scarce. This article aims to present the results of a confirmatory factor analysis and the reliability analysis of the Consequences of Academic Disappointment Inventory. It also aims to provide information on the relationship between academic disappointment and other constructs (i.e., academic motivation, perfectionism, self-critical rumination, and negative emotions).

**Methods:**

The current study is a correlational study with a cross-sectional design, and the data were collected via an online platform. A total of 512 Hungarian students participated in the study. Participants were asked to recall a situation in which they received negative feedback and consequently felt disappointed in the past few months of their academic life. They were then asked to complete a series of questionnaires.

**Discussion:**

The results show that students experience disappointment with themselves, their performance, and the authority giving the feedback in different ways. Self- and performance-related disappointments are similar in terms of their revealed factor structures (i.e., motivation, lack of motivation, behavioral investment, and lack of behavioral investment). Whereas disappointment with the authority has a different factor structure. The current results also show that academic disappointment can be either an activating or a passivating emotion in terms of its effects on student engagement. Our results revealed a set of significant factors associated with students' engagement in the context of academic disappointment, including feelings of shame and hostility, perfectionism, self-critical rumination, intrinsic motivation, extrinsic motivation for external regulation, and amotivation.

**Supplementary Information:**

The online version contains supplementary material available at 10.1186/s40359-025-02610-6.

## Introduction

Academic emotions (AEs) were shown to play a key role in student engagement and success in the past decades [1]. Despite the increasing interest in studying AEs, the research in the field is concentrated on a select few of a wide range of academic emotions, the most prominent example being anxiety [2, 3] whereas other AEs are rather neglected. Current knowledge and research in the literature on academic disappointment (AD) is rather scarce. Academic disappointment, on the other hand, is evidenced to be frequently experienced by students [4, 5]. Indeed, in their education life, students are likely to encounter situations in which they may not attain what they expect to achieve (e.g., a desired midterm grade) [6, 7]. Moreover, AD can lead to a decrease in motivation towards academic life [8, 9]. Acquiring further knowledge about AD, can serve to aid educators and practitioners to better grasp their students` academic experiences and to implement strategies to help them to cope with AD.

In the literature on AEs, the “Control Value Theory” [10] provides an elaborated framework that sheds light on to the relationship between students` emotions, student engagement with academic life and success. In this framework, AD is classified as a negative-deactivating outcome emotion [11]. Hence, according to the Control Value Theory, AD is conceptualized as a negatively valanced feeling that arises as an outcome of an academic experience and that is expected to decrease engagement with academic life and activities. To our knowledge, there are few research conducted on AD and thus, the existing evidence on the AD is insufficient to understand the nature of this AE. One important reason for the lack of knowledge in the field regarding AD is the fact that there is not any standardized instrument to assess AD. To fill this gap in the literature, in an earlier work, the Consequences of Academic Disappointment Inventory (CADI) was created [12]. The CADI serves to assess previous AD that arose because of receiving negative feedback from an authority figure in the educational context. The inventory gives information about the direction of students` disappointment (AD with oneself as a person, with performance and with the person/authority giving feedback), and about how students experience their disappointment in relation to their subsequent engagement with education life (i.e., motivation towards and behavioral engagement with academic activities). In this previous work, the findings regarding the initial psychometrics of the inventory including the results of Exploratory Factor Analysis (EFA) and the reliability analysis were offered. However, there is a need for further validation of the CADI. The current study aims to present results of Confirmatory Factor Analysis (CFA) and to give in detail information about the factor structures, factor loadings and factor correlations. Secondly, we aim to investigate the relationship between AD and other relevant constructs, and hence, to generate information about the convergent validity of the inventory. Finally, we aim to provide our findings about the reliability of the instrument. In this respect, we deem it important to give information about the constructs that were employed in assessing the convergent validity of CADI and their relevance for research on AD.

### Motivation

“ Motivation” is defined by Vallerand [13]^(p42)^ as “The hypothetical construct used to describe the internal and/or external forces that produce the initiation, direction, intensity, and persistence of behavior.” Despite the scarcity of knowledge on AD in the literature, evidence from the decision-making research on emotions showed that negative emotions, including disappointment, may lead to different patterns in relation to motivation [14, 15]. For instance, Zeelenberg and colleagues [16] reported that when someone feels regret, they are motivated to be more engaged to mend the situation. Whereas when an individual feels disappointed, the person tends to avoid both the feeling and the situation that brought about the disappointment. Accordingly, in the field of education, emotions and motivation are acknowledged to have a mutual relationship. [17, 18] Linnenbrick-Garcia [19] theorized that positive feelings tend to boost motivation while negative feelings tend to bring about a reverse effect.

### Perfectionism

Perfectionism is presumed to have two forms that diversify from each other by their effects on a person`s psychological functioning. The first type called “perfectionistic strivings (PS)” refers to seeking perfection and setting very high standards for one`s performance in their pursuit of perfection [20]. The second type named “perfectionistic concerns (PC)” refers to a rather self-critical form of perfectionism that is usually accompanied by self-doubt, fears of being judged and of failing [20]. People who have PS are assumed to have relatively healthy adjustment patterns, whereas people who have PC are thought to have tendency for maladaptive adjustment [21, 22]. It is suggested that perfectionistic people may show a tendency to dwell on thoughts about differences between their ideal and actual states [21, 23]. Taking into account that disappointment is a “negative emotion raised by the divergence between the expected and actual outcome” [24], it is presumed that perfectionism and disappointment may have overlapping aspects.

### Self-critical rumination

Self-critical rumination refers to repetitive and negative thinking characterized by a self-devaluation quality [25]. The self-critical ruminations, just like self-criticism, are assumed to be driven by feeling of shame [25, 26]. Self-criticism, on the other hand, is assumed to have one form that relies on harsh comparisons between oneself and others and another form that relies on negative evaluations about ability to pursue one`s ideals and standards about oneself [27]. In this regard, it is deemed that the experience of AD and self-critical ruminations are similar in the sense that they both rely on a divergence between the expectation and the actual state/situation.

### Negative emotions

Studying emotions in an isolated manner can be challenging at times because it is likely that any given academic situation triggers several emotions with varying intensities at simultaneously. It is also worth mentioning that most emotions may have overlapping elements. For example, both shame [28] and disappointment [24] are supposedly brought about by a divergence between the ideal and actual state. Evidence from cognitive psychology hints to similarities between emotions in terms of their appraisal patterns as well. For instance, in their work on “appraisal patterns of emotions”, Van Dijk and colleagues [29] identified that feeling of disappointment caused by others and feeling of anger can both arise from experiences that are perceived to be beyond ones` control. To be clear, these emotions are reported to be both experienced with a low sense of agency and with a perception of the other person as the cause and the agent of the situation. On the other hand, outcome related disappointment and sadness are reported to be aroused by situations in which the person perceives oneself to be more in control and as having more agency over the outcome.

### The conceptual utility model for the management of stress and psychological wellbeing

Before presenting our results, we remark that it is important to present a theoretical model to explain relationships between the constructs employed in CADI and the variables that were employed in assessing the convergent validity of CADI. The Conceptual Utility Model for the Management of Stress and Psychological Wellbeing (CMMSPW) is a framework that was developed to facilitate predicting extent of emotional “distress” and/or “well-being” of an individual [30]. Relying on the existing empirical findings, the model was created for application in diverse environments (e.g., educational, clinical and/or organizational). The model asserts that a wide range of “predictive” and “mediating” variables, that comprise both context dependent factors and person dependent factors, play a role in determining the psychological state and performance of students [30]. To exemplify, emotions, emotional abilities and regulation skills of students are postulated to be some of the factors that have the potential to determine the level of students` emotional well-being and their performance [31, 32]. Based on evidence from the literature, the authors of the model also noted that perfectionism is among the factors which can have a mediating effect in this interplay [30]. Departing from this theoretical frame, in the current study, academic motivation, perfectionism, self-critical rumination and other negative emotions are considered to be person dependent factors that can be used both in understanding a student`s experience of academic disappointment and in analyzing the convergent validity of CADI.

### Present study

Overall, the current article aims to present the psychometric properties of the Consequences of Academic Disappointment Inventory, including the results for the confirmatory factor analysis and reliability analysis. In addition, it aims to examine the relationship between academic disappointment and other constructs. In regards of the factorial structure of CADI, based on the results from the previous work, we expect the analysis to yield: 1) a 4-factor solution for the SD and the PD subscales and 2) a 2-factor solution for the OD subscale. In the light of the theories and findings from the literature, we believe that motivation, perfectionism, self-critical rumination, and negative emotions are relevant constructs that need to be taken into account and scrutinized in relation to AD, so as to deepen our knowledge of this AE. Hence, we formulated the following hypotheses to investigate relationship between these constructs and the AD: 1) The “academic motivation” construct is expected to correlate with motivation construct employed in the CADI. 2) The “personal standards” construct is expected to correlate with motivation and behavioral investment constructs of the CADI, especially for the SD and the PD subscales. 3) The “personal concerns” construct is expected to correlate with lack of motivation and lack of behavioral engagement constructs of the CADI, especially for the SD and the PD subscales. 4) The “self-critical rumination” is expected to correlate with the lack of motivation and the lack of behavioral engagement constructs of the CADI, especially with the SD and the PD subscales. 5) The “academic disappointment” is expected to correlate with the feelings of sadness, hostility, and shame.

## Method

### Participants

Being a native Hungarian speaking person and being either an undergraduate or a graduate students were the inclusion criteria for participation to the current study. The sample consisted of 512 Hungarian undergraduate and graduate students across different majors. The sample consisted of 93 males, 412 females and 7 participants from other gender (18%, 80% and 1%). The age of the participants ranged from 19 to 53 years (*M* = 23.22, *SD* = 4.54).

### Instruments

*Short Form of the Positive and Negative Affect Scale (I-PANAS-SF):* I-PANAS-SF is a 10-item short version of the original Positive and Negative Affect Scale (PANAS) [33] designed by Thompson [34]. PANAS is a widely used questionnaire with good reliability and validity [34]. The Hungarian version of the original and the short form of the Positive and Negative Affect Schedule (PANAS) has also been demonstrated as a reliable tool [35]. I-PANAS-SF is comprised of two subscales that aim to assess separately positive affect and negative affect. “Determined” and “inspired” are exemplary items from the scale. Participants are asked to read the items and to report “what extent they feel/felt” emotions defined in the items by rating them on a 5-point Likert scale (*1* = very slightly or not at all, *5* = extremely). In line with the objectives of the current study, the negative affect subscale of I-PANAS-SF was employed. The items of the negative affect subscale of I-PANAS-SF are upset, hostile, ashamed, nervous, and afraid. The reliability of the scale was good in our study (α = 0.83).

*Consequences of Academic Disappointment Inventory (CADI):* CADI is a 51 item inventory designed by the authors [12]. The instrument was developed in Hungarian language and is not yet adapted to other languages. The inventory begins with an instruction that asks participants to recall a situation in which they received negative feedback from a professor or a well-respected authority figure and felt disappointment in the last months in their academic life. The CADI is comprised of 3 subscales with 16 items that aim to measure academic disappointment with oneself as a person (SD), with performance (PD) and with the other person/authority giving feedback (OD) (e.g., midterm grade). Before responding to each subscale, participants are asked to rate the intensity of their disappointment (“After this event, I was disappointed in myself.” “After this event, I was disappointed with my performance.” and “After this event, I was disappointed in the person who gave me the feedback.”) over a 7-point Likert scale (1 = Not true of me at all, 7 = Absolutely true of me). Subsequently, participants are asked to respond to items of each subscale over a 7-point Likert scale (1 = Not true of me at all, 7 = Absolutely true of me). The SD and the PD subscales consist of 4 subsets that measure motivation, lack of motivation behavioral investment (BI) and lack of BI (See Supplementary Material 1 for the items). The OD subscale has a different composition. It has two subsets that consist of one positively worded item subset which includes motivation and BI items and of one negatively worded item subset which includes lack of motivation and lack of BI items. The reliability scores for all motivation, lack of motivation, BI and lack of BI subsets were good (respectively α = 0.88, α = 0.90, α = 0.86, α = 0.87 for the SD and α = 0.92, α = 0.87, α = 0.86, α = 0.87 for the PD subscales). The reliability scores for the subscales of the OD were good (α = 0.92 for the positively worded items subscale and α = 0.89 for the negatively worded items subscale).

*Self-Critical Rumination Scale (SCRS):* SCRS is 10 item scale designed by Smart and colleagues [25] to assess rumination, more specifically self-critical rumination. The 10 items are responded over a 4-point Likert scale (*1* = not at all, *4* = very much). “I always seem to be rehashing in my mind stupid things that I’ve said or done” and “Sometimes it is hard for me to shut off critical thoughts about myself.” are exemplary items from the scale. The scale was originally developed in English and has been demonstrated to have good reliability and validity [25]. Previous research with the Hungarian SCRS showed excellent reliability and validity [36]. The reliability of the scale was also excellent in our study (α = 0.91).

*Short Version of Almost Perfect Scale (SAPS):* S-APS is the short version of Almost Perfect Scale [37] created by Rice and colleagues [38] to assess perfectionistic tendencies. The S-APS was originally created in English, and it has been shown to have good reliability and validity [38]. The Hungarian adaptation of the scale, that was conducted with Hungarian high school students, was also demonstrated to have good reliability [39]. The 8-item scale consists of standards (“I try to do my best at everything I do.”) and discrepancy subsets (“I am hardly ever satisfied with my performance”). The items are answered on a 7-point Likert scale (*1* = strongly disagree and *7* = strongly disagree). The reliability scores for both subsets were good (α = 0.85 for the standards and α = 0.82 for the discrepancy subsets) in our study.

*Academic Motivation Scale (AMS-C):* The scale was developed by Vallerand and colleagues [40] to measure student motivation and was adapted to Hungarian by Tóth-Király et al. [41]. The scale was originally developed in French. It was shown to be a reliable instrument for use in different languages (i.e., English [40] and Hungarian [41]). Participants are asked about their reasons for pursuing higher education and are asked to respond to items such as “For the pleasure that I experience in broadening my knowledge about subjects which appeal to me.” or “Because I want to show myself that I can succeed in my studies”. AMS-C is comprised of 28 items which are answered on a 7-point Likert scale (1 = doesn’t correspond at all, 7 = corresponds exactly). The scale consists of Intrinsic Motivation to Know, Intrinsic Motivation towards Accomplishment, Intrinsic Motivation to Experience Stimulation, Extrinsic Motivation-Identification Regulation, Extrinsic Motivation Introjected Regulation, Extrinsic Motivation External Regulation and Amotivation subscales. The reliability scores were good for all the subsets in the current study (respectively α = 0.0.85, α = 0.85, α = 0.88, α = 0.78, α = 0.79, α = 0.82, α = 0.89).

### Procedure

Participation to this research was voluntary, and convenience sampling was employed. The advertisements for the recruitment were made through different channels such as social media, e-mail chains etc. Data was gathered via Qualtrics survey platform between January and April 2021. The participants were first asked to read and approve the informed consent form. After receiving their approval, they were asked to respond to the survey which took 25–30 min to complete. The data collection for the current research was approved by the Institutional Review Board of Eötvös Loránd University and was carried in accordance with the Helsinki Declaration. Informed consent was obtained from all participants in the study.

### Statistical analysis

The current study is a correlational study with a cross-sectional design and the data was collected online. The analyses were run on SPSS 20 and Mplus 8 [42]. We ran confirmatory factor analysis (CFA) to obtain information about the factor structures and the factor loadings of the CADI and to validate the scale. We employed the robust maximum likelihood estimator (MLR) considering its advantage in producing reliable results [43]. To examine the factor structures, we used goodness of fit indices such as Chi-square (χ2) values, Comparative Fit Index (CFI), Tucker-Lewis Index (TLI), Root Mean Square Error of Approximation (RMSEA) and Standardized Root Mean Square Residual (SRMR) in accordance with the guidelines [44]. TLI and CFI values are deemed in the acceptable range if they are approximate to or greater than 0.90. The RMSEA value is suggested to be acceptable if it is equal to or less than 0.08. To acquire information about the internal reliability, the Cronbach's alpha (α) was measured.

To investigate the convergent validity of the CADI, we first ran bivariate correlation analysis. We also used the Multiple Indicators Multiple Causes (MIMIC) model [45] as it relies on regression analysis that are known to generate better estimates. To examine the bivariate correlations and MIMIC models serve to acquire information about relationships between different constructs.

## Results

### Descriptive results

#### Item discrimination, normality, and content validity

None of the items were eliminated in this first step, as all items in the subsets were detected to be within the acceptable range in regards with the standard criteria for item-total correlations (≥ 0.70), Skewness-Kurtosis (± 2) and were determined to be adequate in terms of content validity. Tables 1 and 2.

**Table 1:** Descriptives for the employed scales

	**Mean**	**SD**	**Minimum**	**Maximum**
I-PANAS SFN	2.74	1.14	1	5
SCRS	2.75	0.80	1	4
SAPS-S	5.78	1.04	2	7
SAPS-D	4.57	1.47	1	7
AMS-C IMT	5.34	1.29	1	7
AMS-C IMA	4.60	1.52	1	7
AMS-C IMES	4.09	1.65	1	7
AMS-C IDE	5.46	1.27	1	7
AMS-C INT	4.63	1.61	1	7
AMS-C EXT	5.17	1.47	1	7
AMS-C AMT	2.02	1.25	1	7

*N* = 427*, Missing* = 85*, I-PANAS SFN *Short Form of Positive and Negative Affect Scale Negative Affect Subscale*, SCRS *Self-Critical Rumination Scale*, SAPS-S *Short Version of Almost Perfect Scale Standards Subscale*, SAPS-D* Short Version of Almost Perfect Scale Discrepancy Subscale, *AMS-C *Academic Motivation Scale*, IMT *Intrinsic Motivation to Know Subscale*, IMA *Intrinsic Motivation towards Accomplishment Subscale, *IMES *Intrinsic Motivation to Experience Stimulation*, IDE *Extrinsic Motivation Identified Regulation Subscale*, INT *Extrinsic Motivation Introjected Regulation Subscale*, EXT *Extrinsic Motivation External Regulation Subscale*, AMT *Amotivation Subscale

**Table 2:** Descriptives for the Consequences of Academic Disappointment Inventory Subsets

	**Mean**	**SD**	**Minimum**	**Maximum**
SD Motivation^a^	3.69	1.39	1	7
SD Lack of motivation^a^	4.13	1.61	1	7
SD BI^a^	4.58	1.28	1	7
SD Lack of BI^a^	2.66	1.30	1	7
PD Motivation^b^	3.67	1.42	1	7
PD Lack of motivation^b^	4.16	1.51	1	7
PD BI^b^	4.57	1.28	1	7
PD Lack of BI^b^	3.05	1.37	1	7
OD Positively worded items^c^	3.45	1.42	1	7
OD Negatively worded items^c^	4.15	1.42	1	7

*N* = 322*, SD *Self-Disappointment Subscale, *PD *Performance Disappointment Subscale, *OD *Disappointment with the Other Person/Authority Giving Feedback Subscale, *BI *Behavioral Investment.

^a^Number of missing values is 37.

^b^Number of missing values is 44.

^c^Number of missing values is 85.

## Construct validity

### Confirmatory factor analysis (CFA)

The four-factor solution was revealed to give the most adequate fit for the SD subset (See Table 3). Although a 3-factor model (3 factor-3) also yielded acceptable goodness of fit indices, the 4-factor solution was identified to have a better fit in terms AIC values. The factor loadings for the 4-factor solution ranged from 0.71 to 0.86 (See Table 4).

**Table 3:** Goodness of Fit Information for the Applied Models for the SD Subscale

Model	χ2 (df)	p	CFI	TLI	RMSEA	90% CI	SRMR	AIC
1 factor	565.448 (104)	0.000	0.797	0.765	0.125	0.115—0.135	0.081	14859.110
2-factor (positive & negative worded items)	493.026 (103)	0.000	0.828	0.800	0.115	0.105—0.126	0.078	14752.881
3-factor 1 (positive worded items & LM & LBI)	265.819 (101)	0.000	0.927	0.914	0.076	0.065—0.087	0.058	14453.525
3-factor 2 (negative worded items & M & BI)	448.493 (101)	0.000	0.847	0.818	0.110	0.100—0.120	0.076	14681.303
3-factor 3 (BI + LBI &M &LM)	223.095 (101)	0.000	0.946	0.936	0.065	0.054—0.077	0.048	14391.442
4-factor (M & LM & BI & LBI)	158.792 (98)	0.000	0.973	0.967	0.047	0.033—0.060	0.040	14311.335

*Note. χ2* = Chi-square value, *(df)* = Degrees of freedom*, p* = significance level, *CFI *Comparative Fit Index, *TLI *Tucker-Lewis Index, *RMSEA *Root Mean Square Error of Approximation (RMSEA), *CI *Confidence interval, *SRMR *Standardized Root Mean Square Residual, *AIC *Akaike Information Criterion, *M *Motivation items, *LM *Lack of motivation items*, BI *Behavioral investment items, and *LBI *Lack of behavioral investment items

**Table 4:** Results for CFA Analysis for the 4-Factor Solution of the SD Subscale

**Item no**	**Self-Disappointment Subscale Factors**			
	**Factor 1**	**Factor 2**	**Factor 3**	**Factor 4**
1	**0.80**			
2	**0.79**			
3	**0.83**			
4	**0.79**			
5		**0.84**		
6		**0.77**		
7		**0.84**		
8		**0.86**		
9			**0.82**	
10			**0.81**	
11			**0.71**	
12			**0.78**	
13				**0.73**
14				**0.86**
15				**0.81**
16				**0.77**
**Factor Correlations**				
Factor 1	**-**			
Factor 2	-0.80	-		
Factor 3	0.84	-0.64	-	
Factor 4	-0.69	0.65	-0.88	-
**Reliability**				
Cronbach alpha	0.88	0.90	0.86	0.87

Similarly, the four-factor model yielded the most adequate fit for the PD subset (See Table 5). The factor loadings for the PD subscale ranged from 0.62 to 0.87 (See Table 6.).

**Table 5:** Goodness of Fit Information for the Applied Models for the PD Subscale

Model	χ2 (df)	p	CFI	TLI	RMSEA	90% CI	SRMR	AIC
1 factor	698.897 (104)	0.000	0.758	0.721	0.143	0.133—0.154	0.084	14418.393
2-factor (positive & negative worded items)	508.963 (103)	0.000	0.835	0.808	0.119	0.109—0.129	0.071	14152.740
3-factor 1(positive worded items & LM & LBI)	424.377 (101)	0.000	0.868	0.844	0.107	0.097—0.118	0.070	14047.358
3-factor 2 (negative worded items & M & BI)	381.768 (101)	0.000	0.886	0.864	0.100	0.089—0.111	0.063	13989.220
3-factor 3(BI + LBI &M &LM)	363.011 (101)	0.000	0.893	0.873	0.097	0.086—0.107	0.065	13969.428
4-factor (M & LM & BI & LBI)	204.174 (98)	0.000	0.957	0.947	0.062	0.050—0.074	0.046	13773.539

*Note. χ2* = Chi-square value, *(df)* = Degrees of freedom*, p* = significance level, *CFI *Comparative Fit Index, *TLI *Tucker-Lewis Index, *RMSEA *Root Mean Square Error of Approximation (RMSEA), *CI *Confidence interval, *SRMR *Standardized Root Mean Square Residual, *AIC *Akaike Information Criterion, *M *Motivation items, *LM *Lack of motivation items*, BI *Behavioral investment items, and *LBI *Lack of behavioral investment items

**Table 6:** Results for CFA Analysis for the 4-Factor Solution of the PD Subscale

**Item no**	**Self-Disappointment Subscale Factors**			
	**Factor 1**	**Factor 2**	**Factor 3**	**Factor 4**
1	**0.86**			
2	**0.86**			
3	**0.84**			
4	**0.87**			
5		**0.78**		
6		**0.77**		
7		**0.81**		
8		**0.82**		
9			**0.62**	
10			**0.83**	
11			**0.85**	
12			**0.79**	
13				**0.69**
14				**0.86**
15				**0.81**
16				**0.85**
**Factor Correlations**				
Factor 1	**-**			
Factor 2	-0.79	-		
Factor 3	0.78	-0.62	-	
Factor 4	-0.61	0.80	-0.83	-
**Reliability**				
Cronbach alpha	0.92	0.87	0.86	0.87

For the OD subset, in terms of the fit indices, the CFA analysis revealed similar results for the 2-factor solution, the 3-factor solution when positively worded items grouped together (3-factor 1) and for the 3 factor solution when negatively worded items grouped together (3-factor 2) (See Table 7). Although both of the 3 factor solutions yielded very high correlations among their factors. For instance, for the 3-factor model when positively worded items grouped together, the correlation between 2nd factor and 3rd factor was 1.016. For the 3-factor solution when negatively worded items grouped together, the correlation between the 2nd and the 3rd factor was 0.975. Hence, we decided to continue our analysis with the 2-factor solution. In this model, the modification indices showed a high correlation between the items 4 and 12 and the items 11 and 14. Thus, we specified the model by adding error covariances between these items. Also, the 16th item yielded a very low factor loading (0.27) and the elimination of the item was noted to generate better results. After these model specifications, the 2-factor solution yielded better results in terms of fit indices (χ2 = 202.301, df = 87,* p* = 0.000; CFI = 0.940, TLI = 0.928; RMSEA = 0.075 [90% CI 0. 0.061—0.088]; SRMR = 0.044; AIC = 11,800,783). The factor loadings for the OD subscale ranged from 0.66 to 0.90 (See Table 8).

**Table 7:** Goodness of Fit Information for the Applied Models for the OD Subscale

Model	χ2 (df)	p	CFI	TLI	RMSEA	90% CI	SRMR	AIC
1 factor	427.217 (104)	0.000	0.839	0.814	0.115	0.103—0.126	0.064	13004.745
2-factor 1(positive & negative worded items)	287.025 (103)	0.000	0.908	0.893	0.087	0.075—0.099	0.050	12815.624
2-factor 2 (positive & negative worded items mod spec)	202.301 (87)	0.000	0.940	0.928	0.075	0.061—0.088	0.044	11800.783
3-factor 1(positive worded items & LM & LBI)	287.078 (101)	0.000	0.907	0.890	0.088	0.076—0.100	0.050	12818.565
3-factor 2 (negative worded items & M & BI)	287.403 (101)	0.000	0.907	0.890	0.088	0.076—0.100	0.049	12815.688
3-factor 3(BI + LBI &M &LM)	412.456 (102)	0.000	0.845	0.818	0.113	0.102 – 0.125	0.091	12966.705
4 factor (M & LM & BI & LBI)	257.471 (98)	0.000	0.921	0.903	0.083	0.071—0.095	0.082	12781.271

*Note. χ2* = Chi-square value, *(df)* = Degrees of freedom*, p* = significance level, *CFI *Comparative Fit Index, *TLI *Tucker-Lewis Index, *RMSEA* Root Mean Square Error of Approximation (RMSEA), *CI *Confidence interval, *SRMR *Standardized Root Mean Square Residual, *AIC *Akaike Information Criterion, *M *Motivation items, *LM *Lack of Motivation items*, BI *Behavioral Investment items, and *LBI *Lack of Behavioral Investment items, *Mod spec *Model specification

**Table 8:** Results for CFA Analysis for the 2-Factor Solution of the OD Subscale

**Items**	**Disappointment with the Other Person/Authority Giving Feedback Subscale Factors**	
	**Factor 1**	**Factor 2**
1	**0.89**	
2	**0.82**	
3	**0.79**	
4	**0.77**	
5		** 0.86**
6		**0.69**
7		**0.77**
8		**0.76**
9	**0.85**	
10	**0.74**	
11	**0.66**	
12	**0.67**	
13		**0.90**
14		**0.70**
15		**0.74**
**Factor Correlations**		
Factor 1	**-**	
Factor 2	-0.84	-
**Reliability**		
Cronach alpha	0.92	0.89

## Convergent validity

To examine the convergent validity, we assessed bivariate correlations among the variables (See Supplementary Material 1 Tables 2., 3 and 4), and we applied MIMIC models separately for each subscale (See Tables 9, 10 and 11). It is important to remark that before starting these analyses we computed a total score for the *Intrinsic Motivation to Know, Intrinsic Motivation towards Accomplishment, and Intrinsic Motivation Towards Stimulation* subscales of the Academic Motivation scale because they were identified to have a high correlation (See Supplementary Material 1 Table 8). We labelled this variable as Intrinsic Motivation (IM). Also, as we identified high correlations among some items of the PANAS during the bivariate analysis (See Supplementary Material 1 Tables 9, 10 and 11), we decided to run the MIMIC models by including only the PANAS items that were revealed to have the highest correlations with the rest of the variables.

**Table 9:** Standardized Regression Weights Between Predictors: Self-Critical Rumination Scale, Latent Factors of Short Version of Almost Perfect Scale, Latent Factors of Academic Motivation Scale, Panas Item Hostility, Panas Item Ashamed and the Latent Factors of Consequences of Academic Disappointment Inventory Self-Disappointment Subscale

	**Gender**	**Age**	**SCRS**	**SAPS-S**	**SAPS-D**	**IM**	**IDE**	**INT**	**EXT**	**AMT**	**Hostility**	**Ashamed**
M	0.00	0.02	−0.05	0.15*	−0.00	0.28**	0.00	−0.01	0.07	−0.16	−0.10	−0.11
LM	0.07	0.01	0.18*	0.00	0.04	−0.25**	−0.01	−0.02	0.06	0.26***	0.02	0.21**
BI	0.01	0.00	−0.12	0.30***	−0.14	0.09	−0.07	0.10	0.22*	−0.07	−0.16*	0.00
LBI	0.04	0.16**	0.09	−0.24***	0.13	−0.05	0.01	−0.04	−0.07	0.25**	0.19**	0.03

*Note. N* = *204, M *Motivation, *LM *Lack of motivation, *BI *Behavioral Investment, *LBI *Lack of Behavioral Investment, *SCRS *Self-Critical Rumination Scale*, SAPS-S *Short Version of Almost Perfect Scale Standards Subscale*, SAPS-D *Short Version of Almost Perfect Scale Discrepancy Subscale, *IM *Computed Value for Academic Motivation Scale Intrinsic Motivation Subscales*, IDE *Academic Motivation Scale Identified Regulation Subscale*, INT *Academic Motivation Scale Introjected Regulation Subscale*, EXT *Academic Motivation Scale External Regulation Subscale*, AMT *Academic Motivation Scale Amotivation Subscale

^*^p < 0.05, **p < 0.01, ***p < 0.001

**Table 10:** Standardized Regression Weights Between Predictors: Self-Critical Rumination Scale, Latent Factors of Short Version of Almost Perfect Scale, Latent Factors of Academic Motivation Scale, Panas Item Hostility, and the Latent Factors of Consequences of Academic Disappointment Inventory Performance-Disappointment Subscale

	**Gender**	**Age**	**SCRS**	**SAPS-S**	**SAPS-D**	**IM**	**IDE**	**INT**	**EXT**	**AMT**	**Hostility**
M	0.03	0.04	0.06	0.15*	−0.11	0.32***	−0.00	−0.06	0.07	−0.13	−0.08
LM	0.07	0.00	−0.02	−0.11	0.20*	−0.09	0.10	−0.02	−0.08	0.27**	0.13
BI	−0.01	−0.04	−0.01	0.28***	−0.24*	0.13	−0.13	0.05	0.29**	0.11	−0.18*
LBI	0.11	0.16**	−0.00	−0.29***	0.24*	−0.02	0.07	−0.00	−0.08	0.19*	0.17**

*Note. N* = *201, M *Motivation, *LM *Lack of motivation, *BI *Behavioral Investment, *LBI *Lack of Behavioral Investment, *SCRS *Self-Critical Rumination Scale*, SAPS-S *Short Version of Almost Perfect Scale Standards Subscale*, SAPS-D *Short Version of Almost Perfect Scale Discrepancy Subscale, *IM *Computed Value for Academic Motivation Scale Intrinsic Motivation Subscales*, IDE *Academic Motivation Scale Identified Regulation Subscale*, INT *Academic Motivation Scale Introjected Regulation Subscale*, EXT *Academic Motivation Scale External Regulation Subscale*, AMT *Academic Motivation Scale Amotivation Subscale

^*^p < 0.05, **p < 0.01, ***p < 0.001

**Table 11:** Standardized Regression Weights Between Predictors: Self-Critical Rumination Scale, Latent Factors of Short Version of Almost Perfect Scale, Latent Factors of Academic Motivation Scale, Panas Item Hostility, and the Latent Factors of Consequences of Academic Disappointment Inventory Disappointment with the Other Person/Authority Giving Feedback Subscale

	**Gender**	**Age**	**SCRS**	**SAPS-S**	**SAPS-D**	**IM**	**IDE**	**INT**	**EXT**	**AMT**	**Hostility**
PWI	0.05	−0.01	−0.01	0.04	0.00	0.23**	−0.01	0.05	0.14	0.09	−0.24***
NWI	0.06	0.03	0.07	−0.06	0.02	−0.06	0.01	−0.03	−0.03	0.14	0.33***

*Note. N* = *237, PWI *Positively Worded Items, *NWI *Negatively Worded Items, *SCRS *Self-Critical Rumination Scale*, SAPS-S *Short Version of Almost Perfect Scale Standards Subscale*, SAPS-D *Short Version of Almost Perfect Scale Discrepancy Subscale, *IM *Computed Value for Academic Motivation Scale Intrinsic Motivation Subscales*, IDE *Academic Motivation Scale Identified Regulation Subscale*, INT *Academic Motivation Scale Introjected Regulation Subscale*, EXT *Academic Motivation Scale External Regulation Subscale*, AMT *Academic Motivation Scale Amotivation Subscale

^*^p < 0.05, **p < 0.01, ***p < 0.001

In addition, as we identified that the results of the MIMIC analysis did not vary when controlled for the intensity variable, therefore we decided to eliminate this variable from further analysis. In line with our expectation, MIMIC models showed that motivation and investment had positive correlations with SAPS-Standards in both the SD and PD subsets. Lack of motivation and lack of investment had a positive association with Amotivation in both the SD and PD subsets. Lack of motivation and lack of investment had negative associations with the SAPS-Standards in the SD and PD subsets. Also, the SCRS had no relation with any variables except for the lack of motivation in the SD subset. The IM was revealed to have positive correlations with motivation in the PD subset and with positively worded items in the OD subset while it was revealed to have a negative correlation with lack of motivation in the SD subset. Extrinsic Motivation External Regulation had positive correlations with investment in the SD subscale and with positively worded items in the OD subscale.

For the Panas items, shame was positively associated with lack of motivation in the SD subset. Hostility was correlated negatively with motivation and investment and positively with lack of motivation and lack of investment in both the SD and PD subsets. Hostility was also found to have negative correlation with positively worded items and positive correlations with negatively worded items in the OD subset.

## Discussion

Students encounter various pleasant and unpleasant emotional experiences during their academic life. In turn, the academic emotions are known to affect students’ engagement with their education [10]. Experiencing academic disappointment is a part of students` journey towards learning and personal growth. Scrutinizing AD may serve precious information that can help educators and scientists to better understand students` academic emotions. Also, the acquired knowledge may serve crucial purposes such as prevention of dropouts. However, there are few studies on AD in literature and the lack of a standardized instrument is among reasons for the neglect of the topic. The current research aimed to present our findings regarding the psychometric properties of the Consequences of Academic Disappointment Inventory including the factor structures of its subscales and the factor loadings. Moreover, the current study aimed to inspect the relationships between academic disappointment and other relevant constructs including motivation, perfectionism, self-critical rumination, and negative emotions (e.g., sadness, hostility, and shame). The analysis revealed that the SD and the PD subscales have a 4-factor structure covering motivation, lack of motivation, behavioral investment, and lack of behavioral investment dimensions. The term “motivation” was used to define the first factor as the items that composed this subscale were evaluated to be in essence congruent with the formerly defined motivation construct by Vallerand [13]. The second factor of the SD and the PD subscales were identified to be comprised of the items that designate a lack of motivation. Therefore, this factor was named “the lack of motivation”. The third factor was labelled as “behavioral investment” because this subset was evaluated to mainly consist of the items which indicate concrete engagement in regards of the devoted time and efforts put for academic tasks. As the fourth factor was composed of the items that indicate an absence of behavioral investments, it was labelled as “lack of behavioral investment”. Whereas the OD subscale has a 2-factor structure covering positively worded items and negatively worded items dimensions. According to these findings, the direction of the emotion has an undeniable impact on how the academic disappointment experience is appraised. The evidence also showed that AD can be either an activating or deactivating academic emotion. In regards of the investigated constructs, when a student experience AD, if (s)he also has feelings of hostility or shame, his/her motivation and/or behavior is more likely to be undermined. Intrinsic motivation and having perfectionistic standards are noted to be likely to lead to higher engagement in terms of both motivation and behavioral investment. Interestingly, it is also noted that being externally regulated, regardless of the general motivational state of the student, can lead to higher behavioral investment if the student is experiencing SD or PD. Having perfectionistic concerns, self-critical rumination, and amotivation, on the other hand, were identified to have undermining effects on student engagement.

The findings from the current study confirmed the previously evidenced factor structures of the CADI [12]. In this respect, the results yielded a 4-factor solution for both the SD and the PD subsets of the CADI in line with our expectations. The 4 factors included motivation, lack of motivation, behavioral investment, and lack of behavioral investment. For the OD subset, the findings revealed a 2-factor structure. These results were also in line with our expectations. The 2-factor structure of the OD is comprised of one factor that comprehends items referring to motivation and behavioral investment constructs and another factor that consists of the items referring to lack of motivation and lack of behavioral investment constructs. These results show that a student`s experience of the AD may differ depending upon the target of his/her disappointment. Their experience would have similarities in case if (s)he is disappointed with his/herself or his/her performance. Whereas (s)he would perceive and experience the situation in a different manner if his/her disappointment mainly concerns the other person (i.e., the authority giving the feedback). One reason behind this divergence can be related to how a certain situation is appraised by a person. As mentioned in previous sections disappointment with another person is rather appraised as beyond control while disappointment induced by an outcome is perceived to occur under the control of the subject [29]. The perceived sense of lack of agency and hence, perceiving the other person as the agent who caused the disappointment seems to be an important determinator of the experience. These evidence point to the importance of the social aspect of the situation that caused the disappointment or the AD. Indeed, this type of phenomenological complexity was formulated for other emotions such as shame in the literature [28]. Frijda stated that shame can be triggered either by a difference between the ideal and actual state of the person or by a perceived inability to fulfill or to fit to expectations of other people. Based on the current findings, it is deemed that the difference of the OD subscale from the SD and the PD subscales of the CADI in terms of its factorial structure shows that the academic disappointment can be experienced either with an accent on self-evaluative or social aspect of the situation.

The acquired evidence from the current study also supported the findings from earlier work by showing that academic disappointment is not necessarily a “deactivating” academic emotion in contrary to the existing classification from the Control Value Theory [11]. Students who experience AD seem to vary in their engagement (e.g., motivation or lack of motivation). Although there are very few studies on AD in the literature, some of the existing studies presented findings that support this potential. For instance, Mahfoudh (2017) [46] who investigated emotional responses to feedback in educational settings noted that students who reported to experience AD with received feedback varied in their subsequent engagement with task at hand. While some students used the received feedback to improve their work, some others did not engage at all. Considering that there is scientific evidence on the double-sided nature of other AEs such as shame [47], we believe in the importance of conducting further studies on AD to shed light the nature of this academic emotion.

The bivariate correlations unveiled that hostility was the emotion that was correlated with all the subscales and all of the subsets of the CADI. The results from the MIMIC analysis confirmed these relationships. When controlled for all of the variables, hostility was correlated positively with lack of behavioral investment in the SD and the PD subsets. It was significantly correlated with negatively worded items subset of the OD as well. In line with these findings, it can be interpreted that students can experience AD and hostility concurrently. It can also be suggested that, if students experience SD or PD, and concurrently feel hostility, then they may tend to have lower behavioral investment in their education life. When a student experiences OD and feels concurrently hostile, on the other hand, it may lead to both a lack of motivation and a lack of BI. These results are in agreement with both our expectations, and with the literature. As previously mentioned, anger is an emotional experience in which the person`s sense of agency and of control can become fragile [29]. It is presumed that this perception might be leading to a behavioral inhibition regardless of the motivational state of the person. In the case of OD, this inhibition might be more intensive and might lead to both a motivational and behavioral inhibition as the perceived agent is another person. Therefore, it is believed that the perceived lack of control and agency over the situation causing the AD may bring about a more intensive experience that inhibits students and may lead to low levels of motivation and investment.

The feeling of shame was revealed to be correlated with all of the subsets of the SD subscale in the bivariate correlation analysis. It was also found to be correlated with the lack of motivation subset of the PD subscale and with the negatively worded items subset of the OD subscale. The MIMIC analysis further unveiled that shame is significantly correlated with the lack of motivation subset of the SD subscale. This finding shows that when a student is disappointed, if (s)he feels shame concurrently, (s)he may tend to have low motivation. However, it is an interesting finding that this would not define their behavioral investment. As mentioned in the above sections, feelings of shame and disappointment seem to have similar appraisal patterns as they are both thought to be arise due to a perceived gap between the ideal and actual states of the person [28]. In this case, it can be interpreted that, when a student experiences disappointment with his/herself and concurrently feels shame, the disappointing situation might cause a lack of motivation as the subject (i.e., student) is not content with his/her actual state. Although it appears that this gap between the desired and actual state does not necessarily determine whether (s)he would behaviorally invest or not in his/her education life.

The bivariate analysis` results showed that the Standards subscale of the S-APS is positively correlated with motivation and BI subsets of both the SD and the PD subscales of the CADI. It displayed a negative correlation with the lack of behavioral investment subset of both of the latter subscales. The MIMIC results confirmed these findings. For the OD subscale, the bivariate analysis yielded a significant correlation between the positively worded items (i.e., items referring to motivation and BI) and Standards subscale of the S-APS. And yet, according to the results from the MIMIC analysis there is not any significant correlation between these subscales. For the SD and the PD subscales, these findings are in line with our expectations. They are also in agreement with the previous theories on perfectionism which state that there is an adaptive manner of determining and following standards in seeking perfection about one’s performance [21]. It can be interpreted that students who have personal standards and who experience disappointment with themselves, or their performance are likely to display higher motivation and behavioral investment. However, if students who have personal standards are disappointed with the authority giving the feedback, then their engagement may vary because the OD, in contrary to the SD and the PD, has a social aspect which is likely to cause students to perceive and respond to the academic disappointment from a different angle.

In regards of perfectionistic concerns, the Discrepancy subscale of the S-APS is correlated with lack of motivation and lack of behavioral investment subsets of both the SD and the PD subscales of the CADI according to the findings from the bivariate analysis. For the OD subscale, it was found to be correlated with negatively worded items (i.e., items referring to lack of motivation and lack of BI). The MIMIC analysis confirmed that the Discrepancy subscale was correlated with the lack of motivation and lack of behavioral investment subsets of the PD subscale. This result is partially in line with our expectations. In the previous sections, it was stated that there is a form of perfectionism that is tendent to cause performance anxiety and fear of failure and that can lead to maladaptive functioning [21]. Therefore, it can be suggested that students who have perfectionistic concerns and who are disappointed with their performance on a certain task are likely to lack motivation and lack behavioral investment. This situation might be related to an intense inhibition that avert students from striving to improve their performance because they experienced what they feared and they cannot undo what has happened (i.e., their perceived poor performance). On the other hand, students who have perfectionistic concerns and who experience SD or OD may vary in their engagement if the source of disappointment is either personal issues about oneself or unfulfilled expectations by the other person. And hence, there is potential for change depending upon whether they pursue it or not.

Regarding the self-critical rumination, the bivariate correlation analysis showed that SCRS was correlated with the lack of motivation and the lack of behavioral investment subsets of the SD and the PD subsets. It was found to be correlated with the negatively worded items subset (i.e., items referring to lack of motivation and lack of BI) of the OD subscale. The MIMIC analysis revealed that SCRS was significantly correlated with the lack of motivation subset of the SD subscale. This result is partially in line with our expectations. It is noted that a student is more likely to experience self-critical rumination if the source of his/her disappointment is his/her perception or issues about oneself. Moreover, it can be interpreted that if students who experience SD dwell on self-critical rumination, this may cause them to have a lack of motivation but not necessarily a lack of behavioral investment. This evidence is also in agreement with the literature when considering that self-critical rumination can engender self-devaluing thoughts [25] and, hence, undermine motivation.

Considering correlations between academic motivation and disappointment, the bivariate analysis revealed that Intrinsic Motivation, Introjected Regulation, and Identified Regulation Extrinsic Motivation subscales were correlated positively with the motivation and the behavioral investment subscales of the SD and the PD subsets of the CADI. They were shown to have positive correlations with the positively worded items subset of the OD subscale as well. The amotivation subscale was revealed to be positively correlated with the lack of motivation and the lack of behavioral investment subsets of the SD and the PD subscales of the CADI. Also, it was revealed to be positively correlated with the negatively worded items subscale of the OD subscale. According to results from the MIMIC analysis, when controlled for all the variables, the Intrinsic Motivation is correlated with the motivation subset of the SD and the PD subscales. Also, for the OD subscale, the Intrinsic Motivation was found to have significant correlation with the positively worded items subset. These results are in line with our expectations. As mentioned previously, it is thought that academic disappointment can be either an activating or deactivating emotion depending on different factors such as student characteristics or circumstances surrounding the experience. In case if a student is intrinsically motivated towards their studies, it might be having an effect that facilitates the student to overcome the disappointing experience and to pursue their work [48]. The Extrinsic Motivation External Regulation subscale was shown to correlate with the behavioral investment subsets of the SD and the PD subscales. It can be interpreted that if a student is disappointed with oneself or one`s performance and keep being behaviorally engaged with his/her study independently from his/her motivational state, then his/her behavior may be driven by “rewards and/or constraints”. It is noted that sometimes students may have to pursue their work even if they do not have any desire to do so in order to achieve a certain goal (e.g., completing a course). The results of our analysis revealed that the Amotivation subscale of the AMS-C is positively correlated with the lack of motivation and the lack of BI subsets of both the SD and the PD subscales of the CADI. This finding is in line with our expectations because amotivated individuals are defined to have a low sense of agency and to not to be able to seize connections between their behavior and the outcomes of these behaviors [40]. This perception, on the other hand, is thought to lead to amotivation and to a tendency towards lack of engagement.

This study had several limitations. In the current study, a recall task was employed for event caused disappointment and hence, the study was retrospective. However, recall tasks are widely used in data gathering across different fields as they allow to conduct studies with larger populations [49]. And yet, this method has its flaws. For instance, people`s recollection of their memories may be subject to “recall bias” and the data obtained from participants may be compromised. Additionally, it is possible that certain traits, such as self-critical rumination or perfectionism, may affect the recall of events. Another limitation of the current study concerns the operationalization of the constructs of the CADI. The indicators of the construct of motivation and lack of motivation envisaged in the CADI mainly comprise items that measure either the presence or the lack of a motivation to devote time and effort by students. In this sense, the motivation and the lack of motivation constructs measured by use of the CADI do not comprehend other potentially adaptive or maladaptive forms of motivation. Hence, the operational definitions used in the current study to measure motivation might have led to a limitation in regards of assessment of motivation from a broader perspective. A final limitation concerns the generalizability of the findings. The CADI is originally developed in Hungarian language and was used for data collection in Hungarian student populations. In this sense, the generalizability of the obtained findings to other cultures is not yet possible. There is a need for cultural adaptations of the scale to test its use in international populations and to generalize the acquired evidence.

## Conclusion

The current research aimed to validate the CADI and to inspect relations between academic disappointment and other relevant constructs. In light of the findings from the current study, we conclude that the direction of the AD can be an important factor affecting students` experience of this academic emotion. Students tend to appraise their academic disappointment with themselves, their performance and with the authority giving the feedback in different ways. We also conclude that AD, which was formerly classified as a passivating emotion in the literature [11], can either activate or passivate students in terms of their engagement with education. Academic motivation (i.e., intrinsic motivation, extrinsic motivation for external regulation, and amotivation), perfectionism, self-critical rumination, feeling of hostility and feeling of shame are associated with engagement (i.e., presence or lack of motivation and behavioral engagement) in case of experiencing an AD when receiving negative feedback. These results are also in line with the theoretical frame of the CMMSPW model which suggests that students` emotions and their capacities to regulate themselves during the learning and evaluation process affect their overall psychological state and performance [30, 50]. CADI is a valid and reliable instrument that can be employed in scientific research. It can also be utilized in educational settings by practitioners to identify students who may have decreased engagement (e.g., drop out) when encountering academic disappointment. Prospective research goals may include: 1) To further validate CADI by examining relationships between CADI constructs and other constructs such as “self vs. external regulation” [31] in order to enhance knowledge on individual characteristics that may affect consequences of AD, 2) To employ CADI in experimental studies, 3) To adapt CADI to other cultures.

## Supplementary Information

Additional file 1.

## Data Availability

The databases used and/or analyzed during the current study are available from the corresponding author upon reasonable request.
