# Supplementary material for: Consequences of academic disappointment inventory: confirmatory factor analysis, reliability and convergent validity
Source: BMC Psychol. 2025 Apr 24;13:440. doi: 10.1186/s40359-025-02610-6 (PMC12023593; doi:10.1186/s40359-025-02610-6)
Supplement: Supplementary file 1 — Additional file 1. [file 40359_2025_2610_MOESM1_ESM.docx]

APPENDICES

Table A1.

*Items for Consequences of Academic Disappointment Inventory (CADI)*

| **Item** | **Item content** | |
| --- | --- | --- |
| SD1 | After this event, I was disappointed in myself, but.. | ..I felt motivated to improve. |
| SD2 |  | ..this experience boosted my energy to work. |
| SD3 |  | ..it strengthened my drive to make further efforts. |
| SD4 |  | ..this experience enhanced my motivations. |
| SD5 | After this event, I was disappointed in myself, and.. | ..I felt demotivated. |
| SD6 |  | ..I felt that I have no energy |
| SD7 |  | ..I lost my drive. |
| SD8 |  | ..this experience consumed all my motivations. |
| SD9 | After this event, I was disappointed in myself, but.. | ..I worked harder to be better. |
| SD10 |  | ..I devoted more time to improve myself. |
| SD11 |  | ..I put a lot of effort into becoming a successful person. |
| SD12 |  | ..I followed various opportunities to develop my skills. |
| SD13 | After this event, I was disappointed in myself, and.. | ..I did not do anything about it |
| SD14 |  | ..I did not devote any energy to improve myself. |
| SD15 |  | ..I put no effort into becoming better. |
| SD16 |  | ..I stopped looking for opportunities to develop my skills. |
| PD1 | After this event, I was disappointed with my performance, but.. | ..it motivated me to pursue the task. |
| PD2 |  | ..it boosted my energy to invest in the task. |
| PD3 |  | ..it strengthened my drive to make further efforts on this task. |
| PD4 |  | ..it motivated me to work harder on this task. |
| PD5 | After this event, I was disappointed with my performance, and.. | ..I did not feel like pursuing this task. |
| PD6 |  | ..I felt that I have no energy to devote on this task. |
| PD7 |  | ..I lost my drive to make further efforts on this task. |
| PD8 |  | ..and this experience reduced my motivation to work on this task. |
| PD9 | After this event, I was disappointed with my performance, but.. | ..I planned the necessary steps ahead to achieve my goal in this task. |
| PD10 |  | ..I put a lot of effort into improving my performance. |
| PD11 |  | ..I prepared even more to be successful on this task. |
| PD12 |  | ..I did everything I could to develop my skills on this task. |
| PD13 | After this event, I was disappointed with my performance, and.. | ..I was not invested in becoming successful on this task. |
| PD14 |  | ..I did not put any effort into improving my performance. |
| PD15 |  | ..I stopped practicing for the task |
| PD16 |  | ..I did not devote any time to develop my skills on this task. |
| OD1 | After this event, I was disappointed in the person who gave me the feedback, but.. | ..it motivated me to learn further from him/her. |
| OD2 |  | ..it boosted in me a drive to pursue opportunities for benefiting from his/her knowledge. |
| OD3 |  | ..it enhanced my motivation to work harder so as to receive positive feedback from her/him. |
| OD4 |  | ..it motivated me to devote my energy to change his/her view of me. |
| OD5 | After this event, I was disappointed in the person who gave me the feedback, and.. | ..I lost my drive to learn from him/her. |
| OD6 |  | ..I felt that I have no energy to change his/her view of me. |
| OD7 |  | ..it reduced my motivation to benefit from his/her knowledge. |
| OD8 |  | ..I felt like I did not have any motivation to receive positive feedback from him/her. |
| OD9 | After this event, I was disappointed in the person who gave me the feedback, and.. | ..I pursued opportunities to learn from him/her more. |
| OD10 |  | ..I worked harder so as to gather knowledge by seriously considering his/her opinion. |
| OD11 |  | ..I devoted time and energy to benefit from his/her knowledge. |
| OD12 |  | ..I put a lot of efforts to change his/her view of me. |
| OD13 | After this event, I was disappointed in the person who gave me the feedback, and.. | ..I completely stopped looking for opportunities to learn from him/her. |
| OD14 |  | ..I did not make any effort to benefit from his/her knowledge. |
| OD15 |  | ..I avoided potential interactions with this person. |
| OD16 |  | ..I did not devote energy in gathering information about why (s)he gave this negative feedback. |

*Note.* *SD* = Self-Disappointment Subscale, *PD* = Performance Disappointment Subscale, *OD* = Disappointment with the Other Person/Authority Giving Feedback Subscale.

Table A2.

*Bivariate Correlations between the Factors of Academic Motivation Scale*

|  | **IMT** | **IMA** | **IMES** | **IDE** | **INT** | **EXT** | **AMT** |
| --- | --- | --- | --- | --- | --- | --- | --- |
| **IMT** | 1 | - | - | - | - | - | - |
| **IMA** | .71* | 1 | - | - | - | - | - |
| **IMES** | .72* | .67* | 1 | - | - | - | - |
| **IDE** | .43* | .39* | .25* | 1 | - | - | - |
| **INT** | .26* | .52* | .26* | .32* | 1 | - | - |
| **EXT** | .01 | -.07 | -.14* | .46* | .37* | 1 | - |
| **AMT** | -.45* | -.34* | -.27* | -.37* | .00 | .05 | 1 |

*Correlation is significant at the 0.01 level (2-tailed).

*Note. N= 427, IMT* = Academic Motivation Scale Intrinsic Motivation To Know Subscale*, IMA =* Academic Motivation Scale Intrinsic Motivation Towards Accomplishment Subscale*, IMS =* Academic Motivation Scale Intrinsic Motivation To Experience Stimulation, *IDE* = Academic Motivation Scale Extrinsic Motivation Identified Regulation Subscale*, INT* = Academic Motivation Scale Extrinsic Motivation Introjected Regulation Subscale*, EXT* = Academic Motivation Scale Extrinsic Motivation External Regulation Subscale*, AMT* = Academic Motivation Scale Amotivation Subscale.

Table A3.

*Results for Bivariate Correlations between the Factors of SD Subscale and the PANAS Items*

|  | **Motivation** | **LM** | **BI** | **Lack of BI** | **Upset** | **Hostile** | **Ashamed** | **Nervous** | **Afraid** |
| --- | --- | --- | --- | --- | --- | --- | --- | --- | --- |
| **Motivation** | 1 | - | - | - | - | - | - | - | - |
| **LM** | -.71** | 1 | - | - | - | - | - | - | - |
| **BI** | .72** | -.55** | 1 | - | - | - | - | - | - |
| **Lack of BI** | -.59** | .57** | -.76** | 1 | - | - | - | - | - |
| **Upset** | -.08 | .28** | .01 | .03 | 1 | - | - | - | - |
| **Hostile** | -.16* | .17* | -.20** | .26** | .31** | 1 | - | - | - |
| **Ashamed** | -.15* | .33** | -.08 | .16* | .35** | .12 | 1 | - | - |
| **Nervous** | -.04 | .24** | -.04 | .06 | .56** | .34** | .17** | 1 | - |
| **Afraid** | -.14* | .23** | -.06 | .14* | .10 | .00 | .44** | -.86 | 1 |

*Note. N =* 285*, LM =* Lack of Motivation, *BI =* Behavioral Investment.

*Correlation is significant at the 0.01 level (2-tailed).

**Correlation is significant at the 0.05 level (2-tailed).

Table A4*.*

*Results for Bivariate Correlations between the Factors of PD Subscale and the PANAS Items*

|  | **Motivation** | **LM** | **BI** | **Lack of BI** | **Upset** | **Hostile** | **Ashamed** | **Nervous** | **Afraid** |
| --- | --- | --- | --- | --- | --- | --- | --- | --- | --- |
| **Motivation** | 1 | - | - | - | - | - | - | - | - |
| **LM** | -.71** | 1 | - | - | - | - | - | - | - |
| **BI** | .70** | -.54** | 1 | - | - | - | - | - | - |
| **Lack of BI** | -.57** | .72** | -.72** | 1 | - | - | - | - | - |
| **Upset** | -.03 | .20** | .07 | .00 | 1 | - | - | - | - |
| **Hostile** | -.15* | .22** | -.23** | .25** | .31** | 1 | - | - | - |
| **Ashamed** | -.03 | .18** | .04 | .06 | .35** | .12 | 1 | - | - |
| **Nervous** | -.06 | .21** | .02 | .05 | .56** | .34** | .17** | 1 | - |
| **Afraid** | -.04 | .08 | .01 | .14* | .10 | .00 | .44** | -.08 | 1 |

*Note. N =* 278, *LM =* Lack of Motivation, *BI =* Behavioral Investment.

*Correlation is significant at the 0.01 level (2-tailed).

**Correlation is significant at the 0.05 level (2-tailed).

Table A5.

*Results for Bivariate Correlations between the Factors of OD Subscale and the PANAS Items*

|  | **PWI** | **NWI** | **Upset** | **Hostile** | **Ashamed** | **Nervous** | **Afraid** |
| --- | --- | --- | --- | --- | --- | --- | --- |
| **PWI** | 1 | - | - | - | - | - | - |
| **NWI** | -.76** | 1 | - | - | - | - | - |
| **Upset** | -.04 | .18** | 1 | - | - | - | - |
| **Hostile** | -.24** | .36** | .31** | 1 | - | - | - |
| **Ashamed** | .04 | .14* | .35** | .12 | 1 | - | - |
| **Nervous** | -.08 | .18** | .56** | .34** | .17** | 1 | - |
| **Afraid** | .12 | .06 | .10 | .00 | .44** | -.86 | 1 |

*Note.* *N* = 237, *PWI* = Positively worded items subset, *NWI* = Negatively worded items subset.

*Correlation is significant at the 0.01 level (2-tailed).

**Correlation is significant at the 0.05 level (2-tailed).

Table A6.

*Results for Bivariate Correlations between the Factors of SD Subscale and Other Predictors*

|  | **Motivation** | **LM** | **BI** | **Lack of BI** | **Intensity** | **Gender** | **Age** | **SCRS** | **SAPS-S.** | **SAPS-D.** | **IM** | **IDE** | **INT** | **EXT** | **AMT** |
| --- | --- | --- | --- | --- | --- | --- | --- | --- | --- | --- | --- | --- | --- | --- | --- |
| **Motivation** | 1 | - | - | - | - | - | - | - | - | - | - | - | - | - | - |
| **LM** | -.71** | 1 | - | - | - | - | - | - | - | - | - | - | - | - | - |
| **BI** | .72** | -.55** | 1 | - | - | - | - | - | - | - | - | - | - | - | - |
| **Lack of BI** | -.59** | .57** | -.76** | 1 | - | - | - | - | - | - | - | - | - | - | - |
| **Intensity** | -.03 | .21** | -.01 | .12* | 1^a^ | - | - | - | - | - | - | - | - | - | - |
| **Gender** | -.00 | .10 | .09 | -.05 | .10^a^ | 1^a^ | - | - | - | - | - | - | - | - | - |
| **Age** | .00 | -.00 | .00 | .10 | -.10^a^ | -.13*^a^ | 1^a^ | - | - | - | - | - | - | - | - |
| **SCRS** | -.16* | .36** | -.20** | .24** | .40** | .02 | -.09 | 1 | - | - | - | - | - | - | - |
| **SAPS-S** | .17* | .00 | .24** | -.16* | .16** | .10 | .02 | .16* | 1 | - | - | - | - | - | - |
| **SAPS-D** | -.08 | .25** | -.14* | .19** | .45** | .06 | -.10 | .53** | .43** | 1 | - | - | - | -. | - |
| **IM** | .40** | -.34** | .32** | -.26** | .11 | .14* | .04 | -.09 | .23** | .04 | 1 | - | - | - | - |
| **IDE** | .25** | -.19** | .31** | -.24** | .12 | .02 | .00 | -.03 | .09 | -.04 | .41** | 1 | - | - | - |
| **INT** | .15* | -.06 | .20** | -.09 | .21** | .09 | .03 | .25** | .32** | .24** | .39** | .37** | 1 | - | - |
| **EXT** | .04 | -.01 | .07 | -.02 | .04 | -.01 | .06 | .03 | .07 | -.02 | .04 | .45** | .36** | 1 | - |
| **AMT** | -.36** | .46** | -.32** | .42** | .13* | -.02 | .07 | .33** | -.14* | .21** | -.45** | -.37** | -.07 | .01 | 1 |

*Note.* *N* = 285 , *LM =* Lack of Motivation, *BI =* Behavioral Investment, *SCRS =* Self-Critical Rumination Scale*, SAPS-S =* Short Version of Almost Perfect Scale Standards Subscale*, SAPS-D =* Short Version of Almost Perfect Scale Discrepancy Subscale, *IM =* Computed Value for Academic Motivation Scale Intrinsic Motivation Subscales*, IDE =* Academic Motivation Scale Identified Regulation Subscale*, INT =* Academic Motivation Scale Introjected Regulation Subscale*, EXT =* Academic Motivation Scale External Regulation Subscale*, AMT =* Academic Motivation Scale Amotivation Subscale, ^a^ *N =* 322.

*Correlation is significant at the 0.01 level (2-tailed).

**Correlation is significant at the 0.05 level (2-tailed).

Table A7.

*Results for Bivariate Correlations between the Factors of PD Subscale and Other Predictors*

|  | **Motivation** | **LM** | **BI** | **Lack of BI** | **Intensity** | **Gender** | **Age** | **SCRS** | **SAPS-S.** | **SAPS-D.** | **IM** | **IDE** | **INT** | **EXT** | **AMT** |
| --- | --- | --- | --- | --- | --- | --- | --- | --- | --- | --- | --- | --- | --- | --- | --- |
| **Motivation** | 1 | - | - | - | - | - | - | - | - | - | - | - | - | - | - |
| **LM** | -.71** | 1 | - | - | - | - | - | - | - | - | - | - | - | - | - |
| **BI** | .70** | -.54** | 1 | - | - | - | - | - | - | - | - | - | - | - | - |
| **Lack of BI** | -.57** | .72** | -.72** | 1 | - | - | - | - | - | - | - | - | - | - | - |
| **Intensity** | -.00 | .09 | .06 | .09 | 1^a^ | - | - | - | - | - | - | - | - | - | - |
| **Gender** | .03 | .11 | .08 | .03 | .10^a^ | 1^a^ | - | - | - | - | - | - | - | - | - |
| **Age** | -.02 | .01 | -.03 | .11 | -.10^a^ | -.13*^a^ | 1^a^ | - | - | - | - | - | - | - | - |
| **SCRS** | -.08 | .18** | -.11 | .18** | .40** | .02 | -.09 | 1 | - | - | - | - | - | - | - |
| **SAPS-S** | .17* | -.05 | .19** | -.18** | .16** | .10 | .02 | .16* | 1 | - | - | - | - | - | - |
| **SAPS-D** | -.07 | .20** | -.13 | .18** | .45** | .06 | -.10 | .53** | .43** | 1 | - | - | - | - | - |
| **IM** | .41** | -.27** | .27** | -.22** | .11 | .14* | .04 | -.09 | .23** | .04 | 1 | - | - | - | - |
| **IDE** | .25** | -.21** | .31** | -.20** | .12 | .02 | .00 | -.03 | .09 | -.04 | .41** | 1 | - | - | - |
| **INT** | .14* | -.06 | .16* | -.05 | .21** | .09 | .03 | .25** | .32** | .24** | .39** | .37** | 1 | - | - |
| **EXT** | .03 | .01 | .06 | .00 | .04 | -.01 | .06 | .03 | .07 | -.02 | .04 | .45** | .36** | 1 | - |
| **AMT** | -.34** | .39** | -.18** | .37** | .13* | -.02 | .07 | .33** | -.14* | .21** | -.45** | -.37** | -.07 | .01 | 1 |

*Note.* *N* = 278, *LM =* Lack of Motivation, *BI =* Behavioral Investment, *SCRS =* Self-Critical Rumination Scale*, SAPS-S =* Short Version of Almost Perfect Scale Standards Subscale*, SAPS-D =* Short Version of Almost Perfect Scale Discrepancy Subscale, *IM =* Computed Value for Academic Motivation Scale Internal Motivation Subscales*, IDE =* Academic Motivation Scale Identified Regulation Subscale*, INT =* Academic Motivation Scale Introjected Regulation Subscale*, EXT =* Academic Motivation Scale External Regulation Subscale*, AMT =* Academic Motivation Scale Amotivation Subscale, ^a^ *N =* 322.

*Correlation is significant at the 0.01 level (2-tailed).

**Correlation is significant at the 0.05 level (2-tailed).

Table A8.

*Results for Bivariate Correlations between the Factors of OD Subscale and Other Predictors*

|  | **PWI** | **NWI** | **Intensity** | **Gender** | **Age** | **SCRS** | **SAPS-S.** | **SAPS-D.** | **IM** | **IDE** | **INT** | **EXT** | **AMT** |
| --- | --- | --- | --- | --- | --- | --- | --- | --- | --- | --- | --- | --- | --- |
| **PWI** | 1 | - | - | - | - | - | - | - | - | - | - | - | - |
| **NWI** | -.76** | 1 | - | - | - | - | - | - | - | - | - | - | - |
| **Intensity** | .22** | -.08 | 1^a^ | - | - | - | - | - | - | - | - | - | - |
| **Gender** | .09 | .06 | .10^a^ | 1^a^ | - | - | - | - | - | - | - | - | - |
| **Age** | -.04 | .07 | -.10^a^ | -.13*^a^ | 1^a^ | - | - | - | - | - | - | - | - |
| **SCRS** | .00 | .17** | .40** | .02 | -.09 | 1 | - | - | - | - | - | - | - |
| **SAPS-S** | .14* | -.05 | .16** | .10 | .02 | .16* | 1 | - | - | - | - | - | - |
| **SAPS-D** | .02 | .14** | .45** | .06 | -.10 | .53** | .43** | 1 | - | - | - | - | - |
| **IM** | .31** | -.19** | .11 | .14* | .04 | -.09 | .23** | .04 | 1 | - | - | - | - |
| **IDE** | .23** | -.15* | .12 | .02 | .00 | -.03 | .09 | -.04 | .41** | 1 | - | - | - |
| **INT** | .21** | -.05 | .21** | .09 | .03 | .25** | .32** | .24** | .39** | .37** | 1 | - | - |
| **EXT** | .09 | -.02 | .04 | -.01 | .06 | .03 | .07 | -.02 | .04 | .45** | .36** | 1 | - |
| **AMT** | -.10 | .28** | .13* | -.02 | .07 | .33** | -.14* | .21** | -.45** | -.37** | -.07 | .01 | 1 |

*Note.* *N* = 237, *PWI =* Positively Worded Items Subset, *NWI =* Negatively Worded Items Subset, *SCRS =* Self-Critical Rumination Scale*, SAPS-S =* Short Version of Almost Perfect Scale Standards Subscale*, SAPS-D =* Short Version of Almost Perfect Scale Discrepancy Subscale, *IM =* Computed Value for Academic Motivation Scale Internal Motivation Subscales*, IDE =* Academic Motivation Scale Identified Regulation Subscale*, INT =* Academic Motivation Scale Introjected Regulation Subscale*, EXT =* Academic Motivation Scale External Regulation Subscale*, AMT =* Academic Motivation Scale Amotivation Subscale, ^a^ *N =* 322.

*Correlation is significant at the 0.01 level (2-tailed).

**Correlation is significant at the 0.05 level (2-tailed).
